# White Lead Paint in Erysipelas

**Published:** 1883-06

**Authors:** 


### White Lead Paint in Erysipelas.

Mr. Barwell, of Charing Cross Hospital, in the *Lancet*, March 10th, gives a report of three cases of erysipelas, in which he obtained unusually good results by the application of the old-fashioned remedy, white lead paint. No medicine used, excepting, in some cases, the previous administration of a purge. He applies the paint (made by rubbing up the carbonate of lead with linseed oil, and mixed with turpentine as a dryer) over inflamed skin, and a little beyond it, says that in all cases the pain ceased almost immediately after the application; temperature became normal and a rapid cure resulted. The paint is left on until it gradually comes off with slight desquamation of epidermis. The remedy probably acts by occlusion of air. It may be obtained in any paint shop.—*Cin. Med. News.*

